# Involvement of Tunisian General Practitioners in the Management of Patients at Risk of Infective Endocarditis: A Cross-Sectional Study

**DOI:** 10.1155/2021/5542534

**Published:** 2021-03-24

**Authors:** Malek Lagha, Mehdi Khemiss, Ines Kallel, Asma Araissia, Chems Belkhir, Saida Sahtout, Sana Bagga

**Affiliations:** ^1^Department of Conservative Dentistry and Endodontics, Dental Faculty of Monastir, University of Monastir, Monastir, Tunisia; ^2^Dental Medicine Department in University Hospital Fattouma Bourguiba, University of Monastir, Monastir, Tunisia; ^3^Maxilla Functional and Aesthetic Rehabilitation (LR12SP10), Farhat Hached University Hospital of Sousse, Sousse, Tunisia; ^4^Dental Medicine Department in University Hospital Sahloul (Sousse), University of Sousse, Sousse, Tunisia; ^5^Faculty of Dental Medicine of Monastir, University of Monastir, Monastir, Tunisia; ^6^ABCD F Laboratory of Biological Clinical and Dento-Facial Approach, University of Monastir, Monastir, Tunisia

## Abstract

**Objectives:**

This work aimed to estimate the knowledge and practice of general dentists in the governorate of Manouba regarding the management of patients at risk of infective endocarditis.

**Materials and Methods:**

A survey involving private sector general dentists in the governorate of Manouba was performed. It contained 21 questions, and it was addressed to 111 dentists. Only 82 dentists responded. To carry out the descriptive study, we used the SPSS software version 21.0.

**Results:**

Our results proved the lack of knowledge among dentists in the governorate of Manouba with regard to the management of patients at risk of infective endocarditis. An overestimation of the risk and an overprescription of antibiotic were found in order to ensure an over-protection for the patients as well as the dentist. In fact, 85.4% of these dentists prescribed antibiotic prophylaxis for the two groups of patients (high risk and moderate risk). Only 9.8% followed the right modality of antibiotic prophylaxis prescription; 4.9% of the dentists prescribed antibiotic only 1 hour before the act and 4.9% of them prescribed antibiotic 1 hour before the act and continued the treatment in case of the presence of an infectious site.

**Conclusion:**

A discrepancy towards an over-estimation of risk and overprescription of antibiotic was found between the recommendations and real practice. Similar studies in the other governorates of Tunisia are recommended in order to better understand the problem.

## 1. Introduction

Infective endocarditis (IE) is an uncommon but potentially devastating disease with an estimated annual prevalence of 11–50 cases per million population [1]. In Tunisia, a study conducted by Letaief et al. in 2007 showed an incidence of around 55 cases per million population, stable during 10 years with 46 new cases per year [2]. In a retrospective study carried out by Rim Lakdhar et al., based on the analysis of 135 files of patients hospitalized in the Cardiology Department of The RABTA Hospital during the period from January 1981 to December 2011, the portal of entry was identified in 59 (47.3%) patients and the dental cause was present in 21% of cases [2]. Therefore, the dentist plays a major role in the prevention of IE in patients at risk. The management of the latter has been guided by international consensus since 1995.

Since the publication of the American Heart Association (AHA) in 1990, it has been conventionally considered appropriate to prevent IE by antibiotic prophylaxis (AP) before procedures believed to cause bacteremia [3–5]. However, the evidence supporting the effectiveness of AP was poor, deriving solely from animal studies or case series [4, 6]. These growing doubts led to a major revision of the AHA guidelines in 2007, narrowing the indication for AP to a smaller population of at-risk individuals [4]. In Europe, similar guidelines to those in the US restricting AP to only patients with highest risk of IE were issued in 2009 [3]. In contrast, complete restrictions of AP were placed in the United Kingdom (UK) in 2008 by The National Institute for Health and Clinical Excellence (NICE). They based their decision on the absence of randomized controlled trial assessing the efficacy of AP for the prevention of IE [3, 4, 7].

Recently, a meticulous analysis of epidemiological data by Dayer et al. reported a significant increase of incidence of IE in England (0.11 cases per 10 million people per month) that appeared to correspond with the NICE recommendations to cease AP [8]. NICE guidelines were revised to “antibiotic prophylaxis against infective endocarditis is not recommended routinely for people undergoing dental procedures” [3, 4, 9]. In March 2017, the American Dental Association (ADA) recommended that dental professional continue to follow the AHA guidelines, including the dosage regimens [3, 10].

In our study, we aim to assess the attitude and practice of dentists regarding patients at risk of IE and their way to provide dental care in these clinical situations, through a questionnaire.

## 2. Materials and Methods

A cross-sectional survey was conducted to determine antibiotic prescription modalities among 129 private dentists for general practice in Tunisia (in the governorate of Manouba). The list of dentists practicing in this governorate was provided by the Dental Council, following a prior request. The survey took place over a period of 5 months.

### 2.1. Inclusion/Exclusion Criteria

Our study included general practitioners registered with the Dental Council practicing in the governorate of Manouba. This study does not relate to students with pending thesis, residents registered with the Dental Council, and dental specialists or having had specialized training or practicing in public health.

### 2.2. Pretesting of Questionnaire

A self-administered structured questionnaire was developed and tested among a sample of 20 interns and residents, who were interviewed to gain feedback on the overall acceptability of the questionnaire in terms of length and language clarity (no tests were carried out in order to measure the acceptability of the questionnaire). The final version of the questionnaire was written by a resident. It consisted of 3 sections. The first section solicited general demographics (age, gender, years of practice, university studies). The second section consisted of an assessment of knowledge about different heart diseases and the third section consisted of antibiotic prescription modalities in patients at risk of infective endocarditis. The questionnaire was distributed to the 129 dentists in the sample, as a written document; multiple responses for the same question were allowed. To carry out the descriptive study, we used the SPSS software version 21.0 (Armonk, NY: IBM Corp). Normality was tested by the KS test for age and length of practice. The chi-squared test was used during the bivariate analytical study when comparing the percentages of qualitative variables with a threshold *p* < 0.05 for a significant difference between the variables.

## 3. Results

In the present study, out of 129 dentists, 82 responded to the questionnaire and the response rate was 73.8%. The responses were analyzed as percent scores.

Mean age of the 82 dentists is 39 ± 11 years, the minimum being 27 years and the maximum 65 years. It is made up of 38 (46.3%) women and 44 (53.7%) men; the sex ratio is 1.15. The average length of practice is 11 ± 9.5 years, the minimum being 1 year and the maximum 35 years.

Most of them (72, 87.8%) studied at faculty of dental medicine of Monastir and the 10 (12.2%) others outside the country (Algeria, Ukraine, Romania, Russia, and France).

The assessment of the risk of IE in different types of heart diseases (without risk, moderate risk, high risk), by our sample, is shown in Tables 1–3. Most of the respondents estimated correctly the high risk of IE in patients with mechanical or bioprosthetic mitral valve replacement (80.5%) and patients with history of IE (96.3%) (Table 1). 63.4% of respondents considered valvular heart diseases (a moderate risk of IE) as a high risk of IE (Table 2). Most of our respondents overestimated the risk-free heart diseases (Table 3).

Table 4 displays dental procedures justifying antibiotic prophylaxis in patients at high risk of infective endocarditis. The majority of respondents administered AP before conservative infragingival treatment (84.1%), root separation without periodontal disease (81.7%), tooth extraction (80.5%), subgingival scaling/root planning, and mucosal incision procedures (78%).

Table 5 depicts dental procedures not justifying AP in patients at high risk of IE. The majority of respondents did not indicate AP before procedure; however AP was administered by 42.7% before locoregional/local anesthesia injection and 37.8% before bloodless gingival scaling.

Table 6 depicts dental procedures contraindicated in patients at high risk of infective endocarditis.

Evaluation of antibiotic prescribing patterns is depicted in Table 7. 85.4% of our respondents recommended AP in patients at high and moderate risk of IE. 35.4% of dentists prescribed AP as a flash one hour before the procedure, to be continued regardless of the clinical situation. In case of flash prescription, 54.9% of dentists prescribed Amoxicillin (2 g), whilst 37.1% prescribed Amoxicillin (3 g). In case of allergy, Clindamycin was prescribed by 58% of respondents. 32.9% of dentists respected a 10-day interval between prescriptions.

## 4. Discussion

Certain dental procedures are associated with bacteremia although the magnitude will vary [1]. The number of IE cases which originate from an invasive dental procedure appears to be low with only 2–5% of IE patients having undergone such a procedure in the 3–12 months prior to their diagnosis [11]. Furthermore, a study conducted by Chinedu U. Ugwumba et al. showed that the prevalence of bacteremia associated with tooth extraction after preoperative mouthwash with 0.2% chlorhexidine was significantly lower than when patients had preoperative mouthwash without chlorhexidine [12]. The AHA recommends chlorhexidine or povidone-iodine mouth rinses whilst the British Society for Antimicrobial Chemotherapy (BSAC) recommends the administration of chlorhexidine gluconate mouth washes 0.2% held for 1 min in mouth, before dental procedures to narrow bacteremia [11, 13].

The use of probiotic was proposed to modify the oral environment in recent study. This showed promising results in oral microbiota control for both adults [14] and children [15].

The cumulative exposure to bacteremia is significantly greater from everyday procedures such as tooth brushing and chewing, when compared to dental procedures [1, 11, 16].

Through our study, we tried to assess the estimation of the risk of IE by our respondents. Cyanotic congenital heart diseases were underestimated as a high risk of IE. Most of heart diseases with moderate risk or risk free of IE were overestimated. Our results emphasize the lack of knowledge of the infectious risks relating to the various heart diseases. Our results are similar to those found on survey among dentists in Hyderabad City, India, showing a relatively low level of knowledge of the new guidelines [17].

This can be explained by the following:

1. Lack of medical information regarding heart disease even with the definitions provided during the interview.
2. General practitioners prefer to overestimate than underestimate. The vital prognosis in a private practice constitutes a phobia pushing the general practitioner to overestimate and therefore to protect himself.

In vivo, PA are believed to act by interfering with 3 of the major stages in the pathogenesis of IE: bacteremia (by reducing the numbers of microorganisms in blood), adherence (by decreasing the affinity of microorganisms for heart valves), and multiplication of the microorganisms on heart valves (by interfering with the metabolic activity of the microorganism) [18]. However, The AHA guideline Prevention of Infective Endocarditis (2007) states that “no published data demonstrate convincingly that the administration of prophylactic antibiotics prevents IE associated with bacteremia from an invasive procedure” [3, 4, 6, 19].

In the present study, we tried to assess the attitude of dentists regarding patients at risk of IE induced by dental procedures justifying antibiotic prophylaxis and contraindicated acts in patients at risk of IE. In this regard, we have adopted the classification of ANSM 2011. We were able to draw the following conclusions:

1. For dental acts justifying an antibiotic prophylaxis, there is an incorrect contraindication by these dentists. This is due to the responsibility and the fear felt towards this category of patients: 23.2% contraindicated endodontic treatment in single/double rooted teeth, 20.7% contraindicated single-visit endodontic treatment in teeth with vital pulp and rubber dam in place, and 14.6% contraindicated tooth extraction.
2. For dental procedures that do not justify antibiotic prophylaxis, we note that there is an over-prescription of ATB, particularly for local or locoregional anesthesia in uninfected tissue (42.7%), bloodless scaling (37.8%), and conservative supra-gingival treatment (17.1%).
3. For contraindicated dental acts, we note that there is a remarkable lack of knowledge of these acts and a remarkable over-prescription of ATB: 18.3% do not contraindicate intraligamentary anesthesia with no AP whilst 45.1% indicate AP for this act. 72% of respondents still proceed to root separation with periodontal disease with AP. 40.2% do not contraindicate periapical surgery, with AP. Endodontic retreatment (28%) and implant placement (30.7%) are indicated with AP too.

These conclusions present some disagreement with the new views of experts in 2017 who were part of French Society of Cardiology (ESC), French Society of Stomatology, Maxillofacial Surgery and Oral Surgery (SFSCMFCO), French Society of Periodontology and Oral Implantology (SFPIO), and French Society of Endodontics (ESE). These experts consider that endodontic treatments and implants are no longer systematically contraindicated [20].

Savarrio et al. conducted a study for 30 patients receiving non-surgical root canal treatment, a detectable bacteremia was present in 30% of the patients. In a review of 53 cases of IE following dental procedures, 7 were attributed to previous RCT. Duval et al. concluded that bacteremia result from previous infectious lesions rather than dental care [21]. In all cases, there was clear evidence of extra canal instrumentation [22]. With regard to non-surgical endodontics, the AHA and BCS only recommend AP if root canal instrumentation is beyond the apex [11].

Farrington showed that incidence of bacteremia after pulpotomy is 4%. But if there is no certainty that a bacteremia is induced during RCT, then it would seem unlikely that a pulpotomy procedure should cause microorganisms to enter bloodstream [11, 23].

Roberts stated that the placement of matrix band and wedge resulted in bacteremia comparable with that encountered following dental extraction, thus providing evidence that these procedures should be covered by AP [24]. In 2004, Roberts stated that dental procedures associated with bleeding are no longer exclusively indicated for AP as many procedures cause bacteremia without discernible bleeding. Bacteremia generated during dental procedures usually contain no more than 10ᵌ CFU.mL/10 of blood [11]. This is on contrast to animal studies linking bacteremia and IE, where the concentration of organisms is artificially high and thus extrapolation of experimental animal data to the clinical setting is difficult [4, 6, 11].

The evidence-base for the efficacy of antibiotic prophylaxis in preventing IE is weak and views on the risk-benefit analysis have shifted in recent years, with moves to reduce the utilization of antibiotic prophylaxis [4, 6, 25]. In the absence of a robust evidence, growing doubts to this widely accepted practice led to a major revision of the NICE guidelines: “antibiotic prophylaxis against infective endocarditis is not recommended routinely for people undergoing dental procedures” [3, 4, 7, 26].

In the present study, we noted that 85.4% of respondents prescribe antibiotic prophylaxis for both groups of patients (at high risk and at moderate risk). This over-prevention of IEs is also reflected in the prescription modalities: Only 1/3 of our sample follows the reference modalities: either only 1 hour before the act (4.9%) or 1 hour before the act and continue the prescription of antibiotics in the presence of an associated infectious focus (24.4%).

The 2007 guidelines stated that AP should be administered in a single dose 1h before the procedure [3, 6, 11]. However, special circumstances can arise in clinical practice. For example, in the event of dosage of antibiotic not administered before procedure, it may be administered for to 2h after the procedure. For patients already receiving an antibiotic that is also recommended for IE prophylaxis, then a drug should be selected from another class. In these situations, Clindamycin, Azithromycin, or Clarithromycin would be recommended for AP [3]. Amoxicillin is the drug of choice as AP among Nigerian general dentists (89%); in contrast, general dentists in Japan prescribe Cephem followed by Penicillin [27].

As for the doses indicated, in our study, the percentages are very close. 54.9% prescribe 2g of amoxicillin and 45.1% 3 g of amoxicillin in the absence of penicillin allergy. In attempt to reduce the adverse gastrointestinal effects of high dose, the AHA has revised its recommended oral dose of Amoxicillin from 3 to 2 g [3, 11]. However, they also stated that higher serum concentration of Amoxicillin might be expected in some individuals after 10–12 h. Thus, BSAC and BCS still call for 3 g [11].

In the event of Penicillin allergy, 58% of the respondents prescribe Clindamycin. This molecule is not available on the Tunisian market which leads us to underline the non-application of their theoretical knowledge in the field. Other molecules are prescribed such as Clarithromycin, Rovamycin, Pyostacin, Spiramycin, Birodogyl, and Azithromycin. Apart from Azithromycin and Clarithromycin which can be used according to the AHA 2007 as AP in a single dose 500 mg 1 hour before the act, the other molecules do not have the marketing authorization as AP [15, 28]. Clindamycin is the drug of choice, in case of allergy, among dentists in USA (57,3%) and Spain (65,4%) [29, 30]. If possible, treatment should be delayed until at least 10 days after completion of the antibiotic to allow re-establishment of usual oral flora [3, 31]. In our study, only 32.9% of dentists respected this statement.

Bacterial resistance to antibiotic could be involved in the inefficacy of the prophylaxis [15, 32]. Pasquantinio et al. showed a reduced susceptibility of oral streptococci to Penicillin in 13.4% of cases [33]. Gopalakrishnan et al. underlined that, based on the lack of clear benefit, even theoretical or rare risks like anaphylactic reactions should be factored in when making public health recommendation affecting large patient population [25]. Restricted access to dentistry due to COVID-19 has resulted in increased dental antibiotic prescription across England [34].

For infected teeth in patients at risk of IE, endodontic treatment should be the treatment of choice because extraction has been associated with greater bacteremia provided:

1. Perform an appropriate technique under ideal aseptic conditions
2. Be performed by a specialist who is trained to perform these types of treatment under ideal conditions
3. Perform a CBCT to assess difficulty and feasibility in one session
4. Prescribe a flash ATB
5. Ensure that working length is respected and that the risk of propelling infected debris beyond the apex is low
6. Provide recapitulation and adequate irrigation to reduce bacteremia

## 5. Study Limitations

The size of the population studied does not allow us to represent all practitioners, but it gives an idea of the therapeutic attitudes among general practitioners. This sample is insufficient to carry out an analytical study, which makes our study a descriptive one.

## 6. Conclusions

Our findings revealed a confusion in the indicated and contraindicated acts and showed a gap between recommendations and clinical practice. There are deficiencies in knowledge regarding prescribing antibiotic and appropriate antibiotic prophylaxis.

This study showed the importance of improving knowledge about patients at risk of infective endocarditis and procedures requiring antibiotic prophylaxis.

## Figures and Tables

**Table 1:** Assessment of high-risk heart disease.

	Without risk		Moderate risk		High risk	
	Nb	%	Nb	%	Nb	%
Untreated cyanotic congenital heart disease	8	(9.8)	23	(28)	51	(62.2)
Treated cyanotic congenital heart disease (persistent shunt)	3	(3.7)	29	(35.4)	50	(60.2)
Mechanical or bioprosthetic mitral valve replacement	2	(2.4)	14	(17.1)	66	(80.5)
Patient with history of infective endocarditis	1	(1.2)	2	(2.4)	79	(96.3)

**Table 2:** Assessment of moderate-risk heart disease.

	Without risk		Moderate risk		High risk	
	Nb	%	Nb	%	Nb	%
Non-cyanogenic congenital heart disease	16	(19.5)	41	(50)	23	(30.5)
Mitral valve prolapse	5	(6.1)	38	(45.3)	39	(47.6)
Hypertrophic cardiomyopathy	20	(23.4)	37	(45.1)	25	(30.5)
Patient with stable prosthetic heart valve	25	(30.5)	39	(47.6)	18	(22)
History of myocardial infarction less than 6 months old	6	(7.3)	19	(23.2)	57	(69.5)
Aortic bicuspidia	16	(19.5)	50	(61)	12	(19.5)
Valvular heart disease (aortic insufficiency, mitral insufficiency, atrial stenosis)	3	(3.7)	27	(32.9)	52	(63.4)
Aortic stenosis	21	(25.6)	30	(36.6)	31	(37.8)

**Table 3:** Risk-free heart disease assessment.

	Without risk		Moderate risk		High risk	
	Nb	%	Nb	%	Nb	%
Treated cyanotic congenital heart disease	13	(15.9)	34	(41.5)	35	(42.7)
Patient with pacemaker	43	(52.4)	22	(26.8)	17	(20.7)
History of myocardial infarction older than 6 months	23	(28)	42	(51.2)	17	(20.7)
Coronary artery bypass	29	(35.4)	31	(37.8)	22	(26.8)

**Table 4:** Assessment of dental procedures justifying antibiotic prophylaxis in patients at high risk of infective endocarditis.

	Without flash		With flash		Contraindicated	
	Nb	%	Nb	%	Nb	%
Sub-gingival scaling and root planning	11	(13.4)	64	(78)	7	(8.5)
Conservative infra-gingival treatment	7	(8.5)	69	(84.1)	6	(7.3)
Single visit endodontic treatment in teeth with vital pulp and rubber dam in place	21	(25.6)	44	(53.7)	17	(20.7)
Endodontic treatment in single/double rooted teeth	15	(18.3)	48	(58.5)	19	(23.2)
Mucosal incision procedures (tumor excision, brake-ectomy, etc.)	4	(4.9)	64	(78)	14	(17.1)
Tooth extraction (on arch, entangled, impacted)	4	(4.9)	66	(80.5)	12	(14.6)
Root separation without periodontal disease	5	(6.1)	67	(81.7)	10	(12.2)

**Table 5:** Assessment of dental procedures not justifying antibiotic prophylaxis in patients at high risk of infective endocarditis.

	Without flash		With flash		Contraindicated	
	Nb	%	Nb	%	Nb	%
Bloodless gingival scaling	51	(62.2)	31	(37.8)	0	(0)
Loco regional/local anesthesia injection	45	(54.9)	35	(42.7)	2	(2.4)
Conservative supra-gingival treatment	62	(82.9)	14	(17.1)	0	(0)
Suture removal	69	(84.1)	9	(11)	4	(4.9)
Removable prosthesis placement	75	(91.5)	3	(3.7)	4	(4.9)
Dental X-rays	76	(92.7)	3	(3.7)	3	(3.7)

**Table 6:** Assessment of contraindicated dental procedures in patients at high risk of infective endocarditis.

	Without flash		With flash		Contraindicated	
	Nb	%	Nb	%	Nb	%
Intraligamentary anesthesia	15	(18.3)	37	(45.1)	30	(36.6)
Endodontic treatment in teeth with non-vital pulp	5	(6.1)	29	(35.4)	48	(58.5)
Multi-visit endodontic treatment in teeth with vital pulp	8	(9.8)	30	(36.6)	44	(53.7)
Endodontic retreatment	7	(8.5)	23	(28)	52	(63.4)
Endodontic treatment in multi-rooted teeth with vital pulp	12	(14.6)	39	(47.6)	31	(37.8)
Endodontic treatment without rubber dam	7	(8.5)	31	(37.8)	44	(53.7)
Periapical surgery	1	(1.2)	33	(40.2)	48	(58.5)
Root separation with periodontal disease	4	(2.9)	59	(72)	19	(23.2)
Transplantation	2	(2.4)	16	(19.5)	64	(78)
Replantation	2	(2.4)	16	(19.5)	64	(78)
Implant placement	2	(2.4)	26	(30.7)	54	(65.9)
Bone graft placement	0	(0)	27	(32.9)	55	(67.1)
Periodontal surgery	3	(3.7)	46	(56.1)	33	(40.2)

**Table 7:** Modalities and doses for antibiotic prescription.

			Nb	%
For which group of patients do you recommend antibiotic prophylaxis:	High-risk patients		12	(14.6)
	Both groups (moderate risk and high risk)		70	(85.4)

If the procedure requires antibiotic prophylaxis, how do you prescribe it:	Flash antibiotic prophylaxis (1h before procedure)		4	(4.9)
	Flash antibiotic therapy, to be continued after procedure regardless of the clinical situation		29	(35.4)
	Flash antibiotic therapy, to be continued if focus on infection		20	(24.4)
	Flash antibiotic therapy, to be continued until the treatment is finished		4	(4.9)
	Prescription of antibiotic therapy 2 days before the procedure, to be continued after regardless of the clinical situation		25	(30.5)

In case of flash prescription, which molecule do you prescribe, and at what dose:	Amoxicillin 2g		45	(54.9)
	Amoxicillin 3g		37	(37.1)
	In case of allergy	Clindamycin 600 mg	47	(58)
		Others	34	(42)

A 10-day interval is respected between flashes:	No		50	(61)
	Yes		27	(32.9)

## Data Availability

All data are available within the manuscript with the exception of the questionnaire provided as Supplementary Materials.

## Supplementary Materials

Supplementary Materials A questionnaire was drawn up, intended for general practitioners registered with the Dental Council practicing in the governorate of Manouba, to carry out this study. It consisted of 3 sections. The first section solicited general demographics (age, gender, years of practice, and university studies). The second section consisted of an assessment of knowledge about different heart diseases and the third section consisted of antibiotic prescription modalities in patients at risk of infective endocarditis.
