# Supplementary material for: Involvement of Tunisian General Practitioners in the Management of Patients at Risk of Infective Endocarditis: A Cross-Sectional Study
Source: Int J Dent. 2021 Mar 24;2021:5542534. doi: 10.1155/2021/5542534 (PMC8016554; doi:10.1155/2021/5542534)
Supplement: Supplementary Materials — A questionnaire was drawn up, intended for general practitioners registered with the Dental Council practicing in the governorate of Manouba, to carry out this study. It consisted of 3 sections. The first section solicited general demographics (age, gender, years of practice, and university studies). The second section consisted of an assessment of knowledge about different heart diseases and the third section consisted of antibiotic prescription modalities in patients at risk of infective endocarditis. [file 5542534.f1.docx]

**Involvement of Tunisian general practitioners in the management of patients at risk of infective endocarditis: A cross-sectional study**

**A: General demographics:**

Gender: Female Male
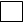


Age: ………….. Years of practice: ……………

University studies: ……………………………………………….

**B: Knowledge:**

1: Among these groups of patients who present a risk of infective endocarditis:

|  | Without risk | Moderate risk | High risk |
| --- | --- | --- | --- |
| Untreated cyanotic congenital heart disease |  |  |  |
| Treated cyanotic congenital heart disease |  |  |  |
| Treated cyanotic congenital heart disease (Persistent shunt) |  |  |  |
| Non-cyanogenic congenital heart disease |  |  |  |
| Mitral valve prolapse |  |  |  |
| Hypertrophic cardiomyopathy |  |  |  |
| Patient with stable prosthetic heart valve |  |  |  |
| Patient with pacemaker |  |  |  |
| Mechanical or bio prosthetic mitral valve replacement |  |  |  |
| History of myocardial infarction older than 6 months |  |  |  |
| History of myocardial infarction less than 6 months old |  |  |  |
| Coronary artery bypass |  |  |  |
| Patient with a history of infective endocarditis |  |  |  |
| Aortic bicuspidia |  |  |  |
| Valvular heart disease (aortic insufficiency, mitral insufficiency,  atrial stenosis) |  |  |  |
| Aortic stenosis |  |  |  |
|  |  |  |  |

2: Faced with these acts for a patient at risk of infectious endocarditis, what do you do?

| Acts | With flash | Without flash | Contraindicated |
| --- | --- | --- | --- |
| Bloodless gingival scaling |  |  |  |
| Sub gingival scaling and root planing |  |  |  |
| Intra ligamentary anesthesia |  |  |  |
| Loco regional/ local anesthesia injection |  |  |  |
| Conservative infra gingival treatment |  |  |  |
| Conservative supra gingival treatment |  |  |  |
| Endodontic treatment in teeth with non-vital pulp |  |  |  |
| Endodontic retreatment |  |  |  |
| Single visit endodontic treatment in teeth with vital pulp and rubber dam in place |  |  |  |
| Multi-visit endodontic treatment in teeth with vital pulp |  |  |  |
| Endodontic treatment in multi-rooted teeth with vital pulp |  |  |  |
| Endodontic treatment in single/double rooted teeth |  |  |  |
| Endodontic treatment without rubber dam |  |  |  |
| Periapical surgery |  |  |  |
| Mucosal incision procedures (tumor excision, brake-ectomy…) |  |  |  |
| Removable prosthesis placement |  |  |  |
| Dental X-Rays |  |  |  |
| Tooth extraction (on arch, entangled, impacted) |  |  |  |
| Root separation without periodontal disease |  |  |  |
| Root separation with periodontal disease |  |  |  |
| Transplantation |  |  |  |
| Replantation |  |  |  |
| Implant placement |  |  |  |
| Bone graft placement |  |  |  |
| Periodontal surgery |  |  |  |
| Suture removal |  |  |  |

**C: Prescription**

1: For which group of patients do you recommend antibiotic prophylaxis?

High risk patients
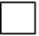


Moderate risk patients
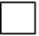


Both groups
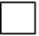


2: If the procedure requires antibiotic prophylaxis, how do you prescribe it?

Flash antibiotic prophylaxis (1h before procedure)
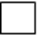


Flash antibiotic therapy, to be continued after procedure regardless the clinical situation
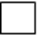


Flash antibiotic therapy, to be continued if focus in infection
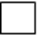


Flash antibiotic therapy, to be continued until the treatment is finished
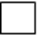


Prescription of antibiotic therapy 2 days before the procedure, to be continued after regardless of the clinical situation
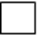


3: In case of flash prescription, which molecule do you prescribe, and at what dose?

Amoxicillin 2g
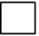


Amoxicillin 3g
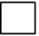


In case of allergy: Clindamycin 600mg
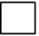


Other: ……………………

4: A 10 days interval is respected between flashes?

Yes
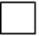


No
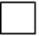


**Thank you!**
